# Lymph node tuberculosis after occupational exposure in a Portuguese physician

**DOI:** 10.47626/1679-4435-2025-1361

**Published:** 2025-06-11

**Authors:** Marta Lagoa, Ana Sofia Ramos, Carlos Ochoa Leite, João Bento, Lisa Pires

**Affiliations:** 1 Serviço de Medicina do Trabalho, Instituto Português de Oncologia do Porto, Porto, Portugal

**Keywords:** tuberculosis, lymph node, occupational diseases, health personnel, occupational health, tuberculose dos linfonodos, doenças profissionais, pessoal de saúde, medicina do trabalho

## Abstract

Tuberculosis is an infectious disease that remains a significant work-related health concern among health professionals. This report describes the case of a 48-year-old immunocompromised physician identified by Occupational Health Services during contact tracing following high-risk exposure to a tuberculosis case. The patient presented with fever, night sweats, and productive cough, and was referred to a Pulmonary Diagnostic Center for further evaluation. After an appropriate investigation, a diagnosis of lymph node tuberculosis was established. Antitubercular therapy was initiated without complications, and the patient returned to work following clinical and radiological improvement. An occupational health assessment was conducted, and a formal report of occupational disease was filed. This case highlights the essential role of Occupational Medicine in the timely identification of workers at risk of exposure and their appropriate follow-up.

## Abstract (pt)

A tuberculose é uma doença infecciosa que continua sendo uma causa importante de agravo relacionado ao trabalho entre os profissionais de saúde. Neste relato, descreve-se o caso de um médico imunodeprimido de 48 anos, identificado pela Medicina do Trabalho para rastreamento de contatos após exposição de risco a um caso de tuberculose. Apresentava febre, hipersudorese noturna e tosse produtiva, sendo encaminhado ao Centro de Diagnóstico Pneumológico para continuação da investigação. Após estudo apropriado, estabeleceu-se o diagnóstico de tuberculose ganglionar. Iniciou-se tratamento antibacilar, sem intercorrências, e o paciente regressou à atividade laboral após melhora clínica e imagiológica, tendo sido avaliado em exame de saúde pela Medicina do Trabalho e efetuado o registro de doença profissional. Neste contexto, destaca-se o papel da Medicina do Trabalho na identificação precoce dos trabalhadores com exposição de risco e na sua subsequente orientação.

## INTRODUCTION

Tuberculosis (TB) is an infectious disease that remains a major cause of morbidity and mortality worldwide. According to the World Health Organization (WHO), it is one of the leading causes of death from a single infectious agent globally.^1^ Despite a declining trend, Portugal continues to have the highest incidence rate of tuberculosis in Western Europe, as reported in the latest Tuberculosis Surveillance and Monitoring Report. In 2020, a total of 1,465 cases were reported in Portugal, corresponding to a notification rate of 14.2 cases per 100,000 inhabitants.^2^

This infection is caused by the *Mycobacterium tuberculosis* bacillus. Transmission occurs primarily through the inhalation of droplets or aerosols containing the bacillus and depends on several factors: the characteristics of the index case (infectiousness), the proximity, frequency, and duration of contact (exposure), the features of the environment where the contact took place, and the characteristics of the exposed individual (individual susceptibility).^3^

TB can affect almost any organ in the body, but the pulmonary form is the most common (69.7% of cases).^1^ Extrapulmonary TB occurs more frequently in immunocompromised individuals, with the lymph node form being the most prevalent. In most cases, it presents as isolated peripheral lymphadenopathy, most commonly involving the cervical chains. The remaining clinical presentation depends on the anatomical region affected and the patient’s immunological status, which makes early diagnosis of extrapulmonary forms more challenging and often requires the use of more invasive diagnostic methods. Lymph node TB is most diagnosed through fine-needle aspiration of the affected lymph node, with identification of the bacillus via culture or direct microscopy and positive nucleic acid amplification testing.^3^

Health professionals are considered a high-risk group for infection with the *Mycobacterium tuberculosis* bacillus. They have a higher risk of exposure to pulmonary TB and a greater incidence of latent infection compared to the general population. This increased risk is attributed to the provision of health care services, which involves closer contact with patients with TB; the performance of certain procedures that may generate or expose individuals to contaminated aerosols in potentially poorly ventilated environments; and inadequate practices, particularly the improper use of personal protective equipment.^4^, ^5^

Decree-Law No. 84/97, of April 16, establishes the protection of workers at risk of exposure to biological agents, including TB, in activities such as those related to hospital services.^6^ Therefore, it is of vital importance that health care institutions implement and maintain robust infection prevention and control strategies aimed at reducing or even eliminating in-hospital transmission and the exposure of health professionals to this biological agent. In this context, Occupational Medicine (OM) plays a critical role in the assessment and management of occupational exposure to TB, thereby ensuring the safety and health of health care workers. This case report highlights the urgency of such an approach by analyzing the case of a physician who contracted the disease while performing professional duties.

## CASE REPORT

We report the case of a 48-year-old male anesthesiologist with ulcerative colitis under biological therapy, identified by OM Services during contact tracing after high-risk exposure to a tuberculosis case. The exposure occurred while providing postoperative care to a bacilliferous tracheostomized patient who had undergone a laryngopharyngectomy, without the use of appropriate respiratory protective equipment. The physician reported previous episodes of high-risk occupational exposure, all with negative screening results and without ever having received chemoprophylaxis.

He presented with a 2-week history of fever, night sweats, and productive cough, with no other associated symptoms. Physical examination revealed no significant abnormalities. Since the exposure context and the onset of symptoms, he was referred to a Pulmonary Diagnostic Center for further evaluation.

Laboratory tests revealed only mild anemia and elevated inflammatory markers. Serologic testing for human immunodeficiency virus (HIV) and sputum mycobacteriologic examination were negative. A chest computed tomography (CT) scan showed multiple lymphadenopathies, particularly in the right paratracheal and subcarinal regions, with no pulmonary parenchymal abnormalities. Given the suspicion of lymph node TB, an endobronchial ultrasound-guided transbronchial needle aspiration of the mediastinal lymphadenopathy was performed, revealing granulomas with caseating necrosis. Direct microscopy, nucleic acid amplification testing, and culture were all positive for *Mycobacterium tuberculosis,* with sensitivity to first-line antitubercular drugs. Upon diagnosis of lymph node TB, the patient began antitubercular therapy, which proceeded without complications.

After 2 months of treatment, at the start of the maintenance phase and following clinical and radiological improvement — evidenced by a reduction in lymph node size — he returned to work. He was subsequently evaluated in an occupational health assessment, and an official report of occupational disease was submitted.

## DISCUSSION

TB remains a significant occupational disease among health professionals. According to the WHO, Brazil ranks 15th among the 22 countries responsible for approximately 80% of global TB cases, with an incidence rate of 45 cases per 100,000 inhabitants — higher than the rate in Portugal, which is below 20 cases per 100,000 inhabitants. In a 2006 study involving 67 health professionals diagnosed with TB, it was found that 42% of the infections were acquired in the hospital setting.^7^ Regarding the extrapulmonary form, a study conducted in Brazil in 2001 with a sample of 930 TB cases found that 17.9% were lymph node TB.^8,9^ In another study conducted between 2002 and 2006, 25 health professionals were identified with TB, among whom 4 (16%) were physicians. The predominant clinical presentation was extrapulmonary in 12 cases (48%), followed by pulmonary TB in 11 (44%), and both forms in 2 cases (8%).^10^ These findings further underscore the importance of accurate differential diagnosis in immunocompromised patients, such as the one described in the present case, where extrapulmonary forms are more common.

This case report highlights the critical role of occupational physicians in the early identification and subsequent management of health professionals exposed to high-risk infectious agents such as *Mycobacterium tuberculosis.* It particularly underscores the importance of implementing and maintaining robust infection prevention and control strategies in health care settings to mitigate disease transmission and ensure the safety of health professionals.

Additionally, it emphasizes the need for coordinated efforts between Occupational Medicine and Pulmonary Diagnostic Centers for effective investigation and management of cases with a probable occupational etiology. The identification and reporting of occupational diseases are essential steps toward improving working conditions and protecting health care staff.

According to the literature, there is a significant degree of negligence among professionals regarding the use of personal protective equipment (with 50% considering it unnecessary). This behavior is associated with factors such as lack of knowledge and habit, discomfort and inconvenience, complacency and forgetfulness due to overconfidence in routine procedures as well as physical fatigue and workload.^11^, ^12^ Adherence to protective measures is closely related to the perception of the risks to which professionals are exposed and their perceived susceptibility.

This case further underscores the importance of raising awareness and providing continuous education for health professionals to promote TB literacy. Emphasis should be placed on recognizing the signs and symptoms of the disease, understanding high-risk activities, and implementing effective prevention strategies — including the correct use of personal protective equipment — while acknowledging the importance of minimizing occupational risks associated with TB exposure.
